# Feasibility and acceptability of an adapted community-based cardiovascular disease prevention program: a pilot study of the StrongPeople strong hearts intervention for women living in urban settings

**DOI:** 10.3389/fpubh.2026.1876157

**Published:** 2026-09-15

**Authors:** Phrashiah Githinji, Rebecca A. Seguin-Fowler, Anita On, Lily Benner, Margaret Demment, Alexandra L. MacMillan Uribe, Jacob Szeszulski, Meredith Graham, Chad D. Rethorst

**Affiliations:** 1 Department of Health and Kinesiology, University of Utah, Salt Lake City, UT, United States; 2 Institute for Advancing Health Through Agriculture, Texas A&M University, College Station, TX, United States; 3 Department of Nutrition, Texas A&M University, College Station, TX, United States; 4 Department of Kinesiology, University of Texas at Arlington, Arlington, TX, United States; 5 Institute for Advancing Health Through Agriculture, Texas A&M University, Dallas, TX, United States; 6 Women, Infants, and Children (WIC) Division, California State Department of Public Health, Sacramento, CA, United States

**Keywords:** cardiovascular disease, nutrition, physical activity, urban, women

## Abstract

**Introduction:**

Black/African American and Hispanic/Latina women living in urban areas are at increased risk of cardiovascular diseases (CVD). Adapting evidence-based interventions (EBIs) to different contexts offers a resource-efficient alternative to developing new interventions. StrongPeople Strong Hearts (SPSH; formerly Strong Hearts, Healthy Communities) is an EBI that significantly improved CVD-related health outcomes and behaviors among rural women. SPSH was adapted for a racially diverse urban female population. Our study examined the feasibility and acceptability of the adapted program to improve behavior and health outcomes.

**Methods:**

We conducted a single-group pre-post pilot study with Black/African American and Hispanic/Latinx women living in an urban area, with a BMI ≥ 25, and engaging in ≤ 150 min of moderate physical activity per week. The 12-week intervention focused on improving diet, physical activity, and CVD prevention skills. We assessed feasibility outcomes (recruitment, attendance, fidelity) and evaluated acceptability through surveys and thematic analysis of interviews.

**Results:**

We enrolled 51 participants; 72.5% (*n* = 37) completed the study Attendance varied across participants, with higher participation observed among completers (median 66.67%) compared to the overall sample (median 54.2%). Participants reported high overall satisfaction (4.89 on a 5-point Likert scale). In interviews, participants (1) reported increased knowledge and skills and improvements in healthy behaviors; (2) praised SPSH implementation including enthusiastic instructors, group support, and structured content, and offered suggestions for enhancing the participant toolkit; (3) appreciated the digital health tools and suggested ways to enhance their use; and (4) expressed desire for more flexible class schedule, extended program duration, and additional content.

**Conclusion:**

Results suggest that the adapted SPSH program is feasible and acceptable among racially diverse women, while additional program refinements (i.e., hybrid program delivery and flexible scheduling) may further enhance program feasibility and acceptability. The desire for digital tools and personalization should be considered in behavior change intervention development.

## 1 Introduction

Cardiovascular diseases (CVD) are the leading cause of death and disability in adult women in the United States, with a disproportionately high prevalence among Black/African American (58.8%) and Hispanic/Latina women (42.7%) (1, 2). These populations also face a greater burden of lifestyle-related CVD risk factors like poor diet and physical inactivity (3, 4). In addition to individual behaviors, social and environmental factors such as social support, food environment, and neighborhood resources impact CVD risk (5–7). Individuals living in urban areas face greater social isolation, less access to healthy foods, and low neighborhood walkability, especially in low-income and minority areas (8–10). As such, evidence-based interventions (EBIs) addressing cultural, contextual, and environmental factors have the potential to address modifiable CVD risk factors.

Adapting existing EBIs to fit specific social and environmental contexts, needs, goals, values, and traditions not only enhances the acceptability and effectiveness of the intervention for diverse populations but also presents substantial advantages in terms of resource efficiency (11, 12). By adapting an established EBI to different settings, instead of developing new programs for each context, we can conserve valuable human and financial resources (11–13). Such adaptations create opportunities to scale the program effectively, extending its reach and impact to broader populations—a critical factor in addressing widespread public health challenges (14). Additionally, these adaptations provide a unique opportunity for comparative analysis. By implementing the program in varied contexts, researchers can gain valuable insights into how different environmental and cultural factors influence the effectiveness of the intervention, guiding future adaptations and improvements (11, 12).

StrongPeople Strong Hearts (SPSH; previously Strong Hearts Healthy Communities (SHHC)) is an evidence-based, multilevel, community-based intervention designed to reduce CVD risk among midlife and older women who are overweight or obese and lead sedentary lifestyles. The program emphasizes behavior modification through experiential learning (15, 16). It focuses on improving dietary intake behavior, increasing physical activity and muscular fitness, achieving weight loss, and imparting other CVD prevention skills and strategies, including stress management (15, 16). Additionally, it seeks to positively affect social and built environments by raising awareness of environmental impacts on health (15).

In two randomized controlled trials conducted between 2015 and 2018 in 16 medically underserved rural communities in Montana and 11 rural towns in New York, SPSH was shown to be effective in improving body weight, body mass index (BMI), diet quality, diet-related psychosocial wellbeing, physical activity, C-reactive protein, and the Life’s Simple 7 CVD risk score among intervention participants compared to control participants (15–17). Furthermore, following a 24-week no-contact period, participants in the intervention group continued to demonstrate improved changes in body weight, blood pressure, dietary patterns, and diet-related psychosocial well-being (18, 19). Given the demonstrated efficacy of SPSH in rural settings (15–17), and recognizing the distinct cultural, contextual, and environmental factors, and specific health needs and goals that influence the health behaviors and outcomes of urban Black/African American and Hispanic/Latina women, we adapted the program, informed by qualitative work with the target population (20), to ensure its relevance and effectiveness within this unique urban context. This current pilot study aims to assess the feasibility and acceptability of the adapted program within an urban and diverse population of Black/African American and Hispanic/Latina women in Dallas, Texas, United States.

## 2 Methods

### 2.1 Study design, participants, and setting

We conducted a single-group pretest-post-pilot trial. Inclusion criteria were: (a) female, (b) self-identified as Black/African American or Hispanic/Latina (c) age 18 and older, (d) having BMI > 25, and (e) engaging in ≤ 150 min per week of moderate physical activity. Exclusion criteria were: (a) pregnant, (b) unable to read and communicate in English, (c) unwilling or unable to provide informed consent, (d) planning to participate in another health behavior change intervention in the next 3 months, and (e) lack of smartphone ownership. This study was approved by the Texas A&M University Institutional Review Board (IRB2022-0801D).

### 2.2 Sample and recruitment

Participants were recruited through a multifaceted approach, including social media advertisements (ads) on Facebook and Instagram, in-person recruitment at various community events, and distributing flyers in high-visibility areas such as community centers/organizations, community health clinics, and bus stops in proximity to our intervention location (12/9/22 to 1/9/23 and 3/22/23 to 4/13/23). Eligible individuals interested in participating in the study were asked to respond to the ads and they completed an online pre-screening questionnaire. The online pre-screening questionnaire consisted of a series of questions assessing eligibility criteria. Those meeting the criteria were then asked to complete an intake form providing contact information to schedule an in-person baseline visit at the West Dallas Multipurpose Center where eligibility was confirmed, pre-intervention data was collected, and participants were invited to participate in the intervention.

### 2.3 Intervention

SPSH is a multi-level program targeting the individual level as well as social and built environments. At the individual level, the emphasis is on behavior modification through experiential learning (15, 16). It is designed to enhance dietary behavior, increase physical activity and muscular fitness levels, promote weight loss, and provide skills and strategies for preventing CVD, including techniques for managing stress (15, 16). The activities included strength training, aerobic exercise, nutrition education, stress reduction activities, and behavior change components like goal setting and health tracking. Additionally, SPSH was designed to influence social and built environments positively by building awareness of how the environment influences health behavior. Activities related to this include grocery store audits and evaluating community food and physical activity environments (15).

Adapting SPSH for a diverse population of urban women involved a stepwise approach guided by the Intervention Mapping (IM) Adapt framework (21, 22). Initially, a needs assessment—details of which are published elsewhere—was conducted to identify the health needs and goals of this population (20). The needs assessment revealed that health programs for Black/African American and Hispanic/Latina women in urban settings should focus on personalized nutrition and practical healthy eating skills and offer accessible and enjoyable exercises (20). Subsequently, the program underwent an evaluation to assess its fit and was modified to better address the identified health determinants, behaviors, and environmental and cultural context of the participants. The curriculum was then revised, a process that involved creating detailed documentation of the changes made to adapt the program. Lastly, plans for implementation and evaluation were formulated, which included the preparation of program materials, training of implementers, and logistical arrangements, as well as the development of evaluation protocols for effective data collection and analysis. The in-depth adaptation process is described in detail in a separate forthcoming publication.

The adapted intervention, initially designed for 24 weeks (48 classes), was re-designed to be delivered twice weekly for 12 weeks (24 classes), with each class scheduled for 1 h, in response to implementation findings from earlier process evaluation studies (23, 24). At the individual level, core areas remained healthy eating, physical activity (aerobic exercise, strength training), and other heart-healthy information and behavioral strategies, including goal setting and stress management. Social and environmental components included peer discussions and involvement of friends and family members in out-of-class activities (e.g., community walks and food environment audits). Program materials and activities were also culturally tailored to better align with the needs and context of Black/African American and Hispanic/Latina urban women. Similar to the second SPSH trial, the adapted program used digital health tools such as Fitbit fitness trackers, Withings smart body weight scales, and exercise videos while continuing to provide other resources such as dumbbells, yoga mats, a participant guide, and a health journal. Participants were encouraged to wear the Fitbit daily for the duration of the study and to use the Withings scale for regular self-weighing. The participant guide included tools to help participants maximize program benefits, such as handouts, takeaway messages, homework, and suggested resources for each class. The health journal was designed to help participants track their dietary habits and physical activity by setting and monitoring goals.

Each class (scheduled for 1 h) was group-based and facilitator-led and combined taught educational content with in-session practice of the target behaviors. Classes included facilitator-led aerobic exercise and strength training, in which participants actively participated, alongside nutrition education delivered through didactic lessons, group discussions, and experiential activities (e.g., food tastings). Goal setting was individually driven within the group format: participants set personal monthly goals and supporting weekly goals, recorded and monitored these in their health journals, and revisited and adjusted them over the course of the program. Stress management techniques were both taught and practiced during class sessions. Self-directed, out-of-class components included homework activities, use of the digital health tools, and activities involving friends and family members (e.g., community walks and food environment audits). Collectively, these activities incorporated behavior change techniques including goal setting, self-monitoring, in-session practice of target behaviors, behavioral feedback, and social support.

All classes were led by the research team (who were also involved in the adaptation process and thus thoroughly familiar with the intervention), and supported by Texas A&M AgriLife Extension agents, and trained Master Wellness community volunteers (25), all of whom have experience delivering health education programs. In addition to their existing expertise, all implementers participated in a comprehensive 3–4 h online leader training course to ensure consistency in delivering the intervention.

### 2.4 Measures and data analyses

*Demographic* data was collected from all participants at baseline using a structured survey capturing key demographic variables (Table 1), and the data was analyzed descriptively. We utilized quantitative assessments to evaluate feasibility outcomes alongside qualitative analyses to understand participant satisfaction and acceptability.

**Table 1:** Participant demographic characteristics.

Characteristic	Mean (*SD*)
Age (years)	45.31 (11.21)
BMI	38.37 (10.43)

	(*n*)	(%)
Race		
Black/African American	28	54.90
Hispanic/Latinx	18	35.30
Mixed/Multiple Race	5	9.80
Ethnicity		
Hispanic/Latina	23	45.10
Not Hispanic/Latina	28	54.90
Education		
Some high school	1	1.96
High school diploma or equivalent GED	7	13.73
Some college	14	27.45
Associate degree	4	7.84
Bachelors degree	16	31.37
Post-graduate degree	9	17.65

*Feasibility* was comprehensively assessed by examining three key aspects: participant recruitment, retention and attendance, and fidelity to the intervention protocol. Participant recruitment outcomes were derived from screening data and enrollment records, while retention was defined as completion of the 12-week follow-up assessment. Attendance was assessed by calculating the proportion of classes attended, based on participant self-check-in forms completed before each session. Fidelity, the extent to which the intervention was delivered as planned, was evaluated through a program fidelity checklist completed by implementers during each class. While one implementer guided the class, another completed the fidelity checklist, which included recording details such as adherence to the planned curriculum, time spent on specific activities including physical activity and nutrition education, and overall class dynamics. The checklist itemized the in-class activities planned for each session as specified in the curriculum, spanning physical activity, nutrition education, and behavioral strategies (e.g., goal setting and stress management); activity completion was calculated as the proportion of these planned activities that were completed as delivered. Fidelity scores can help indicate if the intervention can be delivered as designed but also suggest the overall feasibility of its implementation in an urban setting (26). The quantitative data on feasibility, including recruitment outcomes, attendance, and fidelity scores, were analyzed descriptively. Feasibility and acceptability benchmarks were informed by outcomes observed in prior SHHC trials and process evaluations, including average class attendance of 59.8–67% and delivery fidelity of approximately 81–82% reported in the SHHC process evaluations (23, 24). Feasibility indicators were interpreted collectively rather than judged against any single threshold, consistent with recommendations for pilot and feasibility studies (27).

*Acceptability* was evaluated through a satisfaction survey completed by participants after the 12-week intervention, with items rated on a Likert scale from 1 (Not at all satisfied) to 5 (Very satisfied). Acceptability was further assessed through post-intervention interviews with a randomly selected subsample of participants who completed the intervention (*n* = 19). The interviews were conducted virtually via Microsoft Teams by two research team members (PG and AO) using a semi-structured interview guide (Supplementary File 1) that assessed participants’ experience with different aspects of the intervention. The semi-structured nature of the interviews allowed interviewers to ask probing questions to clarify or get further detail on a participant’s response. Interview questions focused on the participants overall experience in the program, barriers to participation, opinions on nutrition and physical activity components of the intervention, and perceived changes in knowledge, motivation, and behavior, aligning with the social cognitive theory foundation of the SPSH program (28). In line with this theoretical foundation, the interview questions were designed to explore constructs including motivation and self-efficacy for behavior change, self-regulation (e.g., goal setting and self-monitoring through the health journal), and social influences on behavior change (e.g., support from friends, family, and fellow participants). The interviews lasted approximately 35 min each. All interviews were recorded on Microsoft Teams, and the transcriptions were generated using the Teams transcription feature. Immediately after, transcriptions were read and cleaned by the interviewers. We employed a rapid thematic analysis approach due to time and resource constraints. This method facilitates timely data analysis and knowledge synthesis while maintaining rigor and validity, particularly when researchers are trained and experienced in qualitative analysis (29, 30). In our case, all research team members are experienced in qualitative methods. To enhance rigor in our qualitative analysis, we incorporated analyst triangulation involving multiple researchers in the coding and analysis process. This allowed for diverse perspectives and reduced individual bias. Initially, the researcher (AO) summarized the transcripts in a template of key domains developed based on the interview guide. This process involved extracting key ideas and patterns and generating summaries into a Microsoft Excel–based matrix. Subsequently, another researcher (PG) synthesized these by grouping relevant coded text sections and analyzing for emergent themes. Data saturation was reached when no new themes emerged from the interviews. We also engaged in consensus-based interpretation, where the rest of the research team reviewed and verified the validity of the themes, an approach used to substantially enhance the reliability of findings by achieving a consensus on the interpretations (31). The reporting of the study follows the TREND checklist for Improving the Reporting Quality of Nonrandomized Evaluations of Behavioral and Public Health Interventions (Supplementary File 3), while qualitative component of this study follows the Consolidated Criteria for Reporting Qualitative Research (COREQ) checklist (32, 33) (Supplementary File 2).

## 3 Results

Fifty-one participants were enrolled in the study. The participants’ demographic characteristics including age, BMI, race, ethnicity and education level are detailed in Table 1. A total of 51 participants were included in the study, with a mean age of 45.31 (SD 11.21) years and a mean BMI of 38.37 (SD 10.43). The participants were predominantly Black/African American (54.90%) and non-Hispanic/Latina (54.90%), with a significant portion holding a bachelor’s degree or higher (49.02%).

### 3.1 Intervention feasibility

#### 3.1.1 Recruitment

Recruitment for the study involved both online and offline methods, with all participants required to complete an online pre-screening process. Initially, online social media ads were disseminated over a period of 54 days while offline recruitment occurred concurrently. In total, 281 individuals completed the initial screening, and 94 met the eligibility criteria based on the initial screening. Of these, 52 attended the baseline visit, provided informed consent, and underwent eligibility verification. At this point, one participant was found to be ineligible due to a BMI below 25, leaving a final enrollment of 51 participants.

Of the 51 enrolled participants, 40 (78.4%) had been recruited through online social media ads, while 11 (21.6%) were recruited through offline methods, including community health clinics (2 participants), community centers/organizations (2 participants), and previous study participation (1 participant). Additionally, word-of-mouth referrals from participants initially recruited via online ads led to 6 more participants enrolling in the study. Figure 1 provides a detailed overview of the multi-stage recruitment process and the most prevalent reasons for ineligibility.

**Figure 1:**
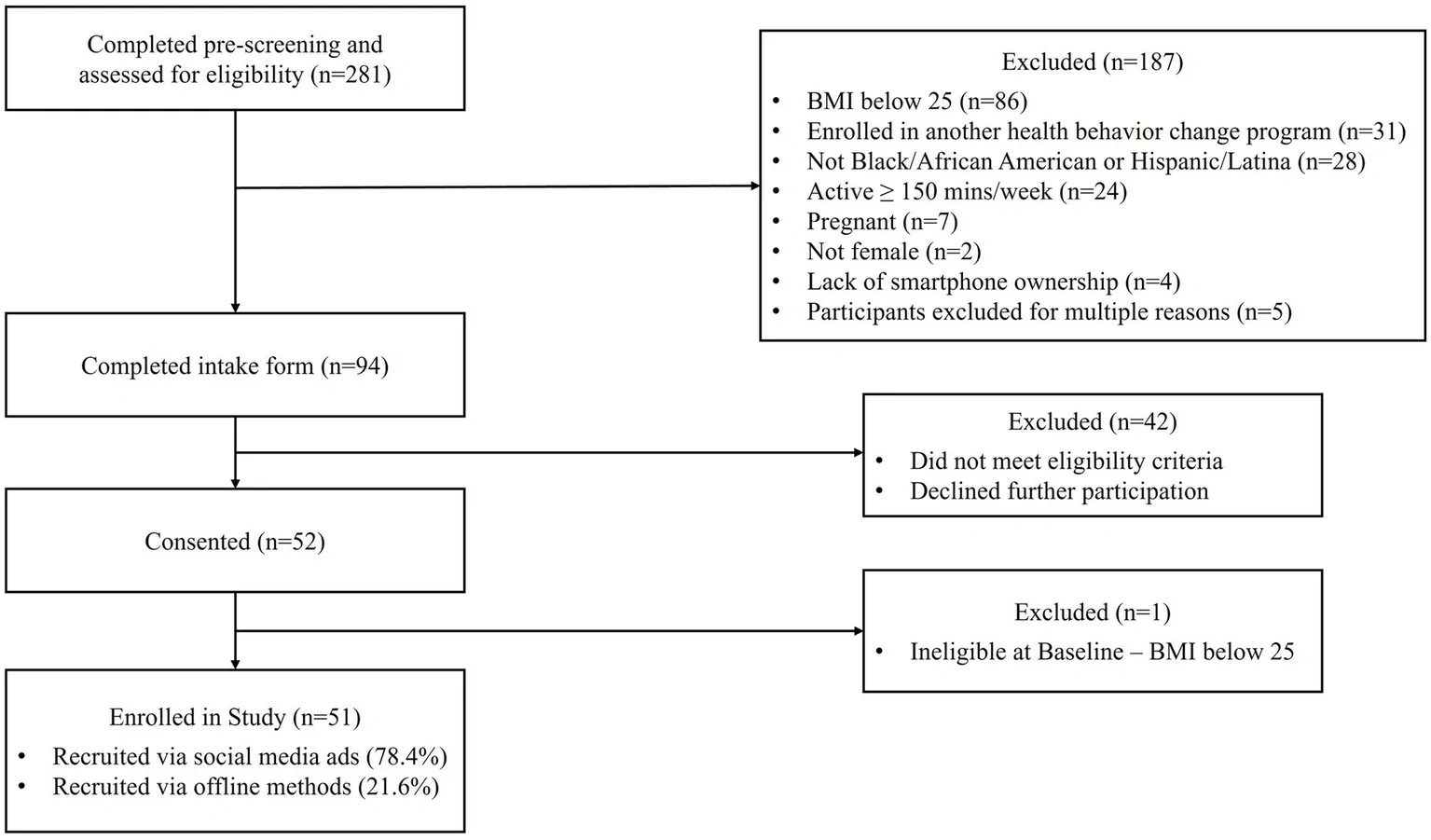
Flowchart of participant screening and enrollment.

#### 3.1.2 Retention and attendance

Study retention at the 12-week end-point visit was 72.5% (*n* = 37). Six participants voluntarily withdrew from the study for various reasons, including schedule conflicts (*n* = 4), a move out of the area (*n* = 1), and feeling uncomfortable in a class setting (*n* = 1). One additional participant completed the baseline data collection but did not attend any class, and seven other participants were lost to follow-up. Attendance varied across participants; median attendance was 54.2% overall and higher among completers (66.67%). Across all participants, median class attendance was 54.2%, while the median class attendance was 66.67% among the “completers” who finished both the intervention and follow-up assessments (*n* = 37; Table 2).

**Table 2:** Comparison of actual versus planned delivery of physical activity in the intervention.

Physical activity (PA)	Actual PA (minutes)	Planned PA (minutes)	Proportion of planned time delivered (%)
Total aerobic time	231	265	87.2%
Total strength time	259	210	123.3%
Total in-class PA	490	475	103.2%

#### 3.1.3 Intervention fidelity

High fidelity was observed, with 82.6% of planned in-class curriculum activities completed and 93.35% of classes delivered as planned. Unforeseen circumstances (power outage, weather) impacted three classes (6.25%), but the content was consolidated to maintain dosage. The average class length was 53.14 min. Participants engaged in a total of 490 min of physical activity during in-class time, averaging 23.33 min per class (Table 3). This included an average of 11 min on aerobic activities and 12.33 min on strength training during each class. Comparing actual versus planned time provides insights into the fidelity of the intervention. Aerobic activities were delivered at 87.2% of the planned time, while strength training time exceeded the plan (123.3%). Overall, the total in-class physical activity delivered slightly exceeded the planned time, totaling 103.2%.

**Table 3:** Comparison of actual versus planned delivery of nutrition education and other related topics in the intervention.

Nutrition education (NE) and CVD-related topics	Actual (minutes)	Planned (minutes)	Proportion of planned time delivered (%)
Total NE time	366	466	78.5%
Total time on other CVD-related topics	260	304	85.5%
Total time	626	770	81.3%

On average, a significant portion of each class (29.81 min) was dedicated to nutrition education and other CVD-related topics (e.g., goal setting and stress management). In total, participants engaged in these topics for 626 min across all classes, less than the planned 770 min (Table 4). The average preparation time required for each class was estimated at 38.3 min. Table 4 provides a detailed breakdown of the actual time spent on each category compared to the planned time.

**Table 4:** Follow-Up Satisfaction Survey (*n* = 37). Items rated on a scale from 1 = Not at all satisfied to 5 = Very satisfied.

Question	Mean score (1–5)
Program satisfaction and acceptability	
Overall satisfaction	4.89
Enjoyability: Lessons	4.92
Class discussions	4.87
Food experience/tastings	4.89
Strength training	4.74
Aerobic exercise	4.87
Perceived behavior-change outcomes	
More physically active	4.05
Physically stronger	4.24
More energy	4.05
Sleep better	3.81
Eating more healthy foods	3.97
Overall health improvement	4.24

### 3.2 Intervention acceptability

Most participants expressed high levels of satisfaction, with an average score of 4.89 out of 5 for the overall satisfaction item (Table 4). This was consistent across various aspects of the program, including the enjoyability of lessons, discussions, food experiences, strength training, and aerobic exercises, which all scored high (satisfaction items: range 4.74–4.92). Participants also reported perceived improvements in lifestyle changes, such as increased physical activity and strength, improved energy levels, healthier eating habits, and overall health (perceived-impact items: range 3.81–4.24). All participants (100%) reported they would recommend the intervention to others.

### 3.3 Qualitative findings on SPSH acceptability

The following themes related to the acceptability of the SPSH-adapted program were identified: (1) the intervention provided participants with knowledge and skills and facilitated positive changes toward healthy behaviors and improved health outcomes; (2) participants praised the SPSH implementation, particularly the instructors, group dynamic, social support, and program structure and they also offered valuable suggestions for enhancing the participant toolkit; (3) participants appreciated the program’s use of digital health tools and also suggested ways to enhance their effective use; and (4) participants expressed a desire for more flexible scheduling of classes, extended program duration, and additional content to accommodate their needs and preferences.

#### 3.3.1 The intervention provided participants with knowledge and skills and facilitated positive changes toward healthy behaviors and improved health outcomes

The intervention led to improvements in participants’ knowledge and skills, fostering positive attitudes that translated into healthier behaviors. Specifically, participants reported gaining skills in reading food labels, developing a greater awareness of their food choices, and adopting healthier eating habits such as incorporating more fruits and vegetables and practicing portion control. One participant shared, *“I learned a different way to think about eating and not just trying to pick food based on how I’m feeling but to put more thought into it” (Participant 11).* Another participant stated, *“I’m now watching my portion sizes, looking at the back at the nutrition labels and looking at that sodium and fat and making sure that I’m eating the correct portion size” (Participant 12).*

Many participants also mentioned increased physical activity levels and improved management of chronic conditions such as blood pressure. For instance, one participant noted, *“It kept me aware of physical activity. I became more mindful of doing physical activity every day, or at least like four days a week” (Participant 1).* This increase in activity not only improved overall well-being but also had tangible health benefits, as another participant shared, *“I’ve been doing regular physical activity for these last 12 weeks. I’m not as winded, my energy is better, and my blood pressure is better” (Participant 13).*

#### 3.3.2 Participants praised the SPSH implementation, particularly the instructors, group dynamic, social support, and program structure and they also offered valuable suggestions for enhancing the participant toolkit

The program received positive feedback, especially regarding its instructors, group dynamics, and social support. Many participants highlighted the instructors’ enthusiasm and genuine interest in their success as critical factors in creating a positive and supportive atmosphere, with one participant noting, *“The best thing about the program was the instructors. They set the tone, with their attitude, enthusiasm, and willingness to be there and seemed genuinely interested in our success” (Participant 10).* The sense of community and accountability fostered within the group setting also motivated participants. One participant shared, *“For me, the group setting is always very beneficial because I just work better in groups when it comes to health. I’m not the most active person; therefore, it gives me motivation when I’m in a group setting” (Participant 5).* Further, the structured schedule of classes provided accountability, as one participant stated, *“There was accountability because I knew I was going to be there every Wednesday and Saturday” (Participant 7).*

The program structure, including fitness sessions, recipes, food tastings, and nutrition education, was popular among many participants and facilitated positive changes. Participants appreciated the structured approach to activities, such as incrementally introducing physical activities to ease integration into their routines. One participant remarked, *“I liked the way the activity was introduced, kind of like in increments because then it made it a lot easier to make it part of a routine” (Participant 4).* Additionally, the breakdown of nutrition content throughout the classes was well-received, as it helped alleviate the overwhelming nature of dietary changes. As one participant noted, “*I thought the way it [nutrition content] was broken down throughout the classes was helpful because it can be so overwhelming. So, to just like go piece by piece.was nice and helpful” (Participant 15).*

Moreover, participants found the in-class food demonstrations and tastings particularly beneficial for expanding their culinary skills and adopting healthier eating habits. One participant appreciated the variety of recipes and the opportunity to taste new foods, stating*, “You gave us a lot of great recipes. I liked the in-class food demos and tasting things. I thought that was great!” (Participant 7).* Another participant highlighted how the tasting sessions encouraged them to try new foods differently, stating, *“There was a tasting in the class, and it made it easier for me to be more open to try things differently. I would have never thought to make my own healthier salad dressing if I had not tasted it in the class” (Participant 10).*

The feedback on the health journal and participant guide provided by the program offered valuable insights for future iterations. While some individuals found them useful for their intended purposes, others expressed a preference for digital health tools over traditional paper formats. One participant who found value in the journal explained, *“The health journal was useful because if you write it down [goals], you can go back and refer to it, tweak it if you need to make changes to move from one milestone to the next or give yourself additional time if you do not meet the milestone” (Participant 10).* Other participants expressed a preference for digital health tools, with one person stating, *“I did not need a book [health journal] that big. I could’ve just used a tablet-based approach” (Participant 11).* Similarly, feedback on the participant guide indicated it was overly detailed and dense, suggesting a need for a more streamlined and accessible approach. One participant shared, *“The workbook [participant guide] was a lot. When you look at it, it was just a lot of words. It was very dense. I do not think I will ever read it outside of class. Maybe there’s a way to pare it down to the most important or the key takeaways” (Participant 1).*

#### 3.3.3 Participants appreciated the program’s use of digital health tools and also suggested ways to enhance their effective use

Most participants appreciated the digital health tools (Fitbit activity tracker and Withings scale) used in the intervention. They expressed positive views about their use in increasing awareness of their health behaviors. For instance, one participant stated, *“It was a physical reminder that I needed to keep moving” (Participant 5).* Another participant highlighted the positive impact of the Fitbit on sleep habits, noting, *“I have always known that I do not sleep well, but I worked actively trying to achieve my sleep score for the Fitbit. I did end up improving my sleep because I was more aware of the sleep score” (Participant 5).*

Some participants encountered challenges with the technology. For instance, one participant experienced difficulties with the weight scale readings. This participant described having issues with fluctuating readings, stating, “*I had trouble with the scale reading. At first, it was fine, but then later I would stand on it, and it would keep fluctuating. I would be as still as I could be, but it would not work” (Participant 15).* Additionally, while some participants appreciated the option to use Fitbit devices to track physical activity, they suggested additional guidance on using it, stating, *“I love the fact that you have that option. However, not everybody may know how to use the fitness component of it—maybe having a class where you guys talk about how to use the Fitbit” (Participant 7).*

#### 3.3.4 Participants expressed a desire for more flexible scheduling of classes, extended program duration, and additional content to accommodate their needs and preferences

Participants consistently expressed a desire for greater flexibility and customization within the program’s format to accommodate their individual needs and preferences better. Specifically, participants expressed a need for more flexible class times, including options for early morning, weekend, and virtual sessions to accommodate various work and personal commitments. For instance, one participant highlighted the challenge of scheduling makeup classes due to limited time slots, stating, *“You know, I wanted to make up for a day. I really could not because the only other class that was offered was during the daytime and not after hours. Which I could not do because I was working” (Participant 6).*

Participants also suggested extending the program duration or incorporating follow-up components to sustain the momentum and support gained during the intervention. One participant said, *“Just when I was getting into a routine, the program ended. I wish there was a way to keep the momentum going” (Participant 13).* Others echoed this sentiment, advocating for longer class durations for deeper engagement and continuous skill development. For example, one participant expressed, *“If I could change something about the program is that I would want it to be for a longer period” (Participant 12),* while another stated, *“I would have liked to have had more time doing aerobics. Maybe 30 min of aerobics and 15 min of strength training. The class should probably have been like 2 h or an hour and a half” (Participant 13).*

Moreover, some participants called for the inclusion of specific content areas, particularly in nutrition education. For instance, one participant stated, *“I would have liked to learn about the various types of food and their nutrients such as the specific vitamins in vegetables and fruits” (Participant 3).* Another participant expressed a desire for broader discussions on food options and greater inclusion of diverse and culturally relevant perspectives within the program, stating, *“I wish there could have been more talk about food options like those found in everyday life, including a greater variety of ethnic cuisines” (Participant 12).*

Additionally, participants suggested that classes scheduled toward the end of the program that did not include physical activity or food-tasting activities could be made virtual. *“Towards the end, when we were not doing exercises or having a tasting, it could have been a Zoom call. I think it’s possible to have some of the classes be virtual because there are a couple I did not think we needed to be there in person” (Participant 1).*

## 4 Discussion

Results of the current study support the feasibility and acceptability of the adapted SPSH intervention for Black/African American and Hispanic/Latina women living in urban areas. Assessing the feasibility and acceptability of lifestyle interventions for CVD prevention in at-risk women provides valuable insights for refining and adapting EBIs before large-scale efficacy trials and broader implementation and scaling-up efforts are undertaken. This approach supports the recommendations of previous research, which highlights the role of pilot and feasibility studies in advancing incremental science through testing and refinement of interventions, to ensure that scaling up health programs is based on robust evidence (34).

Intervention feasibility, including recruitment, retention, and class attendance, was in line with prior studies. Although various recruitment methods were employed, social media emerged as the most effective method for reaching and enrolling our population of interest, accounting for 78.4% of all enrolled participants. While the success of social media advertising in this study highlights its potential for reaching and enrolling Black/African American and Hispanic/Latinx urban women, it is important to note that the overall effectiveness of online recruitment methods can vary. A systematic review by Brøgger-Mikkelsen et al. (2020) found that online recruitment in clinical trials was superior in regards to time efficiency and cost-effectiveness compared with offline recruitment (35). In contrast, a systematic review by Ellington et al. (35) of studies focused on nutrition, physical activity, or obesity-related interventions found that traditional recruitment methods were more effective in recruiting participants than online methods. This divergence in findings could be attributed to the nature of the studies reviewed by both studies, with Ellington et al. focusing on intervention studies with in-person components, while Brøgger-Mikkelsen et al.’s review focused on clinical trials that were fully virtual (36). Additionally, it is important to note that the effectiveness of various recruitment methods can vary depending on factors such as location (e.g., urban vs. rural), age, gender, and the specific online platform used (37–39). Our study contributes to this ongoing dialog by highlighting that the effectiveness of social media recruitment may vary significantly depending on the study’s design and the characteristics of the population of interest.

Study retention at the 12-week end-point visit was 72.5%. A meta-analysis of behavior change interventions found average retention rates in trials of behavior change interventions to be approximately 80% (40); however, retention rates can vary based on context and participant characteristics (41). Furthermore, conduct of feasibility trials should be viewed as an iterative process with the understanding that learnings from the pilot trial will inform refinements to be made prior to launching a larger trial (42). The median attendance was 66.7%, %, comparable to the average attendance reported in the original 24-week SPSH program (67%) and the SHHC-2.0 program (59.8%) (23, 24). We note that the original SPSH program resulted in significant improvements in CVD risk factors.(15-17)Therefore, while attendance observed in this pilot study suggests an opportunity for improvement, the dose of intervention received by participants is likely sufficient to improve CVD risk outcomes. Some reasons for non-attendance or withdrawal included scheduling conflicts, relocation, family and work obligations, and loss to follow-up. Factors such as time and cost constraints, logistical burdens, adverse weather, and competing priorities often impact attendance for in-person behavior change programs (43). These findings highlight the need for strategies to improve engagement and consistent participation in future iterations of the program. Several studies have suggested ways to improve attendance in such programs, including frequent contact attempts by highly skilled staff, providing flexible scheduling options, offering childcare support, having free and accessible transportation, or utilizing virtual sessions (20, 43, 44). Participants in our study echoed several of these strategies, particularly flexible scheduling and virtual delivery of selected components, which are discussed further below.

Assessing fidelity as a measure of understanding the program’s feasibility provides valuable insights into the program’s implementation in a new setting and population. The adapted program demonstrated high fidelity, with 82.6% of activities completed and 93.4% of classes delivered as planned. This fidelity is slightly higher than prior SHHC program and aligns closely with other similar behavioral and lifestyle interventions for adults (24, 45). This indicates strong adherence to the curriculum and successful implementation in an urban setting with diverse participants. However, it is important to note that this level of fidelity may change if the program were to be delivered by individuals other than research staff.

Many participants advocated for a longer program duration, indicating a desire for extended engagement. Moreover, the variability in program engagement raises questions about lifestyle interventions’ optimal duration and frequency. As noted by Voils et al., there is no discernible pattern to suggest an optimally effective intervention period (46). While longer programs or more frequent sessions might be expected to have a greater impact, this may not necessarily be the case (47). For instance, a systematic review found similar effect sizes for both shorter (≤ 6 months) and long-term (≥ 12 months) duration lifestyle interventions (48). This variability underscores the complexity of determining the optimal dose for behavioral interventions.

Additionally, feedback from some participants on having some virtual sessions suggests the potential benefits of leveraging digital solutions to support in-person classes by providing supplemental online content and ensuring the availability of resources for the long-term maintenance of healthy behaviors (49). Many participants advocated for a longer program duration, and they also expressed an appreciation for in-person activities like physical activity and food tastings. However, acknowledging the potential limitations of relying solely on in-person interactions, and integrating virtual sessions could offer a flexible solution. A hybrid model could incorporate in-person exercise classes alongside virtual sessions that provide supplemental online content, such as educational materials or discussions on relevant health topics. As suggested by participants, virtual classes could also be offered when group-based activities, including physical activity, are not scheduled. This approach would not only cater to those who requested a longer program duration but also offer a sustainable avenue for continued engagement and support even after the formal intervention ends. These suggestions are in line with other studies that have demonstrated the advantages of hybrid healthcare approaches. For instance, research conducted in adults in Texas-based Federally Qualified Health Centers (FQHCs) showed that hybrid healthcare models could help bridge racial and ethnic disparities in access to care (50). Black and Hispanic older adults were significantly more likely to have digital program-only visits compared to in-person only visits, highlighting the importance of digital programs as an accessible alternative. However, there were no significant racial and ethnic differences in hybrid utilization, suggesting that hybrid models could serve as an effective strategy to ensure equitable access to health services across different racial and ethnic groups (50).

The adapted SPSH program demonstrated strong acceptability in post-intervention survey results, which revealed high overall satisfaction scores. Participants reported notable lifestyle changes, including improved dietary habits and increased physical activity. These changes were accompanied by perceived improvements in health outcomes, further underscoring the program’s favorable reception. Several key elements emerged as vital contributors to participant engagement and satisfaction. Participants praised the program implementers, describing them as enthusiastic, welcoming, and a big source of motivation. They also valued the program’s structured approach to activities, particularly the incremental introduction of physical exercises and the breakdown of nutrition content. Additionally, the group-based format fostered a strong sense of support and accountability, based on shared health goals. This is also consistent with findings from the original SPSH program, where camaraderie, peer accountability, and exposure to new foods were among the most enjoyed aspects (24). While one participant opted to discontinue due to the group-based class format, the approach resonated with nearly all participants, aligning with our prior needs assessment that highlighted the desire for social support based on shared health goals (20). Furthermore, this group-based approach aligns with established research demonstrating the efficacy of group-based activities in supporting behavior change and improving cardiovascular health compared to individual interventions for addressing CVD risk factors like diet and physical activity (51, 52). For instance, participation in group-based physical activity interventions has been found to result in higher levels of exercise adherence and program compliance across various populations, including minority groups (53, 54). Interpreted through the lens of social cognitive theory, the value participants placed on group support, peer accountability, enthusiastic instruction, and shared health goals reflects the theory’s emphasis on social influences on behavior change, while their appreciation of the incremental structuring of activities is consistent with the creation of mastery experiences, a primary source of self-efficacy (28).

Participants in this study appreciated using digital health tools for health tracking. Still, some participants faced challenges with digital health tools and suggested the need for enhanced support and training in using these digital health tools. It’s important to acknowledge that while technology presents challenges, it also offers significant benefits, such as increased accessibility, real-time tracking, personalization, motivation, and data visualization, which can empower participants to manage their health effectively (55). Therefore, offering more support and training in utilizing these digital health tools could enhance participants’ experience and maximize the potential benefits of technology in promoting health outcomes.

In addition, participants expressed a desire for more discussions about food options, specifically a greater variety of ethnic cuisines. Adaptations were made to include culturally relevant foods, but participants sought even more variety. This feedback highlights the importance of a broader range of culturally diverse foods in health education programs which may enhance the relatability and acceptability of the program. These findings also highlight the dynamic and evolving nature of cultural relevance. Our adaptation process involved a thorough needs assessment and the incorporation of culturally specific dietary considerations; however, the diversity within cultural groups can be vast, and individual preferences and expectations may vary widely. Furthermore, within any broad ethnic category, there are numerous subcultures with distinct dietary preferences (56). The process of cultural adaptation is iterative and ongoing engagement with participants can provide valuable insights for continuous improvement (57).

### 4.1 Study limitations

The study focused exclusively on Black/African American and Hispanic/Latina women in an urban setting, with almost half being highly educated. This focus restricts the applicability of the findings to other demographic groups, and individuals residing in different geographic regions or with differing educational or cultural contexts. We employed a multifaceted recruitment strategy, but ultimately a majority of the participants were enrolled through social media channels, potentially introducing a selection bias and limiting the generalizability of our findings to populations less engaged with these platforms. Moreover, we used self-reported measures, including post-intervention satisfaction surveys and interviews, to assess program feasibility and acceptability. These methods, while valuable, can be susceptible to self-report bias. Furthermore, given that program implementers conducted interviews, there is a risk of social desirability bias. Nonetheless, using both quantitative and qualitative approaches provided a comprehensive understanding of participants’ experiences and perceptions and offered valuable insights into the feasibility and acceptability of the adapted program. Finally, we were unable to collect qualitative feedback from non-completers and feedback from those individuals could provide further insight on refinement of the intervention to increase feasibility and acceptability.

## 5 Conclusion

This study suggests that the adapted SPSH program is acceptable to Black/African American and Hispanic/Latina women in an urban setting, supporting the adaptation of cardiovascular health EBIs to address disparities among diverse populations. Feasibility metrics were largely in line with other behavior change interventions within similar contexts and populations, while feedback from participants can inform further refinement to the program and study procedures to that may further improve retention and attendance. Future research should build upon these findings, investigating strategies to further optimize participant recruitment, retention, and attendance in CVD prevention programs, including exploring the effectiveness of flexible scheduling options, a combination of in-person and virtual components, and user-friendly digital health tools with adequate training and support. Researchers should also examine how to best personalize content and leverage the group-based format to enhance social support and accountability for CVD prevention. Similar programs can leverage these findings by considering strategies to improve program adherence and accessibility, such as implementing hybrid models that integrate online content supplements with traditional in-person sessions. These findings also underscore the potential of social media for recruiting minority populations in urban areas and highlight the need for policies that promote equitable access to digital health tools and provide the necessary training and resources to ensure their effective use. From a policy perspective, adapting EBIs like SPSH may be a feasible, acceptable, and potentially resource-saving approach to address cardiovascular health disparities among diverse populations.

## Data Availability

The raw data supporting the conclusions of this article will be made available by the authors, without undue reservation.
