# Supplementary material for: Feasibility and acceptability of an adapted community-based cardiovascular disease prevention program: a pilot study of the StrongPeople strong hearts intervention for women living in urban settings
Source: Front Public Health. 2026 Sep 15;14:1876157. doi: 10.3389/fpubh.2026.1876157 (PMC13619592; doi:10.3389/fpubh.2026.1876157)
Supplement: Supplementary file 1 [file Data_Sheet_1.DOCX]

**Interview Guide**

**SH Dallas**

*My name is __________, and I am from Texas A&M Agrilife Research in Dallas. Thank you for taking the time to participate in today’s interview. We are interested in learning more about your experience in the Strong Hearts program. This interview will take approximately 30 minutes. As a reminder, we will be recording this discussion. You may skip any questions that you do not wish to answer.*

**Topic 1: Overall Strong Hearts Program**

During this interview we would like you to think about the program classes and not about data collection related activities such as assessment days or recruitment. (Only read probes, which are denoted with letters, if needed)

1. Tell me about your overall experience in the Strong Hearts program for the last three months.
   a. What did you like most about the program?
   b. What did you like least about the program?
   c. If you could make changes to the program, what would be the major things that you would change?
   d. Was the program what you expected?
   e. Was there something you wanted to learn but did not?
   f. What would have made participating in the program easier?
   g. By participating in the SH program, have you become more motivated to maintain or make new lifestyle changes? If so, please explain how.
   h. What changes have you made? In what ways are you different now from when you started? How do you feel about the changes?
   i. What will help you maintain these changes? What will help you not only maintain but also continue to improve your health?
   j. Did the behavioral change strategies help you in making changes?
   k. Would you recommend SH to your friends and family? Tell me a little bit about your response.
      i. Probe any comments that allude to the fit between their culture and the program.
2. Tell me about your experience with the physical activity part of the program.
   a. How did the strength training go?
   b. What did you think of the aerobic exercise components?
      i. Be sure to talk about both at-home and in-class exercise
   c. Did you attend the rec center visit? If so, what did you think?
   d. Did you feel like you had all the resources/tools to do exercises at home? If yes, what were they? If not, what could have been helpful?
   e. What did you think of the Fitbits?
   f. What did you think of the scales?
   g. How useful was the health journal in tracking your goals? Why was or wasn’t it useful to you?
      i. Probe about goal-setting
3. Tell me about your experience with the food and nutrition part of the program.
   a. What did you think went well related to food and nutrition in the program?
   b. What did you learn that you have been able to incorporate into your daily life?
   c. What do you think needs improvement related to food and nutrition?
   d. What did you think of the in-class food experiences? How useful were the in-class food experiences in reinforcing the food and nutrition information you learned?
4. Tell me about your friends and family’s reaction to your participation in the Strong Hearts program.
   a. What things about yourself, your social circle, or your community were you made aware of through your participation in the Strong Hearts program?
   b. Tell us about the friendships you have made during the program.

**Conclusion**

Thank you for all of your time and effort during this program. Is there anything else you would like to share that we didn’t cover?

Thank you very much for your time!
