# Supplementary material for: Feasibility and acceptability of an adapted community-based cardiovascular disease prevention program: a pilot study of the StrongPeople strong hearts intervention for women living in urban settings
Source: Front Public Health. 2026 Sep 15;14:1876157. doi: 10.3389/fpubh.2026.1876157 (PMC13619592; doi:10.3389/fpubh.2026.1876157)
Supplement: Supplementary file 2 [file Data_Sheet_2.docx]

**Supplementary File 2**

**COREQ (Consolidated Criteria for Reporting Qualitative Research) Checklist**

*Based on: Tong A, Sainsbury P, Craig J. Consolidated criteria for reporting qualitative research (COREQ): a 32-item checklist for interviews and focus groups. International Journal for Quality in Health Care. 2007;19(6):349-357.*

| **No.** | **Item** | **Guide question/description** | **Reported (response / manuscript location)** |
| --- | --- | --- | --- |
| **Domain 1: Research team and reflexivity** | | | |
| 1 | Interviewer/facilitator | Which author/s conducted the interview? | Two research team members (PG and AO) conducted all interviews. |
| 2 | Credentials | What were the researcher’s credentials? | PG: PhD; AO: MS. |
| 3 | Occupation | What was their occupation at the time of the study? | Members of the study research team. |
| 4 | Gender | Was the researcher male or female? | Both interviewers were female. |
| 5 | Experience and training | What experience or training did the researcher have? | All research team members are experienced in qualitative methods. |
| 6 | Relationship established | Was a relationship established prior to study commencement? | Yes; interviewers were members of the research team that delivered the intervention, so participants were familiar with them through the program. |
| 7 | Participant knowledge of the interviewer | What did participants know about the researcher? | Participants knew the interviewers as members of the research team delivering and evaluating the program. |
| 8 | Interviewer characteristics | What characteristics were reported about the interviewer/facilitator? | Interviewers were research team members experienced in qualitative methods. |
| **Domain 2: Study design** | | | |
| 9 | Methodological orientation and theory | What methodological orientation was stated? | Rapid thematic analysis (Nevedal et al. 2021; Taylor et al. 2018); interview guide informed by Social Cognitive Theory (Bandura 2004). (Methods) |
| 10 | Sampling | How were participants selected? | Random selection from participants who completed the intervention. |
| 11 | Method of approach | How were participants approached? | Participants were contacted by phone and/or email following random selection. |
| 12 | Sample size | How many participants were in the study? | n=19 interviewees (subsample of 37 completers). |
| 13 | Non-participation | How many people refused to participate or dropped out? Reasons? | None; no selected participants refused to participate. |
| 14 | Setting of data collection | Where was the data collected? | Virtually, via Microsoft Teams. |
| 15 | Presence of non-participants | Was anyone else present besides the participants and researchers? | No one else was present during the interviews. |
| 16 | Description of sample | What are the important characteristics of the sample? | Demographic characteristics of the study sample are reported in Table 1. |
| 17 | Interview guide | Were questions, prompts, guides provided by the authors? Was it pilot tested? | Semi-structured interview guide provided as Supplementary File 1; probing questions were used. The guide was not pilot tested. (Methods) |
| 18 | Repeat interviews | Were repeat interviews carried out? | No repeat interviews were conducted. |
| 19 | Audio/visual recording | Did the research use audio or visual recording to collect the data? | All interviews were recorded on Microsoft Teams. |
| 20 | Field notes | Were field notes made during and/or after the interview? | No field notes were taken; all interviews were recorded and transcribed. |
| 21 | Duration | What was the duration of the interviews? | Approximately 35 minutes each. |
| 22 | Data saturation | Was data saturation discussed? | Yes; saturation was reached when no new themes emerged from the interviews. |
| 23 | Transcripts returned | Were transcripts returned to participants for comment and/or correction? | Transcripts were not returned to participants. |
| **Domain 3: Analysis and findings** | | | |
| 24 | Number of data coders | How many data coders coded the data? | Two primary analysts (AO summarized transcripts; PG synthesized themes), with analyst triangulation involving multiple researchers. |
| 25 | Description of the coding tree | Did authors provide a description of the coding tree? | Transcripts were summarized in a template of key domains developed from the interview guide, generating a Microsoft Excel–based matrix. |
| 26 | Derivation of themes | Were themes identified in advance or derived from the data? | Both: template domains were derived from the interview guide (deductive), and emergent themes were derived from the data (inductive). |
| 27 | Software | What software, if applicable, was used to manage the data? | Microsoft Teams (recording and transcription); Microsoft Excel (analysis matrix) |
| 28 | Participant checking | Did participants provide feedback on the findings? | Findings were not returned to participants for feedback. |
| 29 | Quotations presented | Were participant quotations presented to illustrate the themes/findings? Was each quotation identified? | Yes; illustrative quotations are presented in the Results and identified by participant number. (Results) |
| 30 | Data and findings consistent | Was there consistency between the data presented and the findings? | Yes; themes are supported by quotations, and consensus-based interpretation with the wider research team was used. |
| 31 | Clarity of major themes | Were major themes clearly presented in the findings? | Yes; major themes are presented with supporting quotations in the Results. |
| 32 | Clarity of minor themes | Is there a description of diverse cases or discussion of minor themes? | Yes; divergent perspectives (e.g., preferences regarding the group format and requests for additional device guidance) are reported. |
